# Gender Differences in the Relation Between Suicidal Risk and Body Dissatisfaction Among Bariatric Surgery Patients: A Cross-Lagged Analysis

**DOI:** 10.3390/healthcare12242524

**Published:** 2024-12-13

**Authors:** Gil Goldzweig, Sigal Levy, Shay Ohayon, Sami Hamdan, Subhi Abu-Abeid, Shulamit Geller

**Affiliations:** 1 School of Behavioural Sciences, The Academic College of Tel Aviv-Yaffo, Tel-Aviv 6818213, Israel; giligold@mta.ac.il (G.G.); samihamd@mta.ac.il (S.H.); shulamit@mta.ac.il (S.G.); 2 Statistics Education Unit, The Academic College of Tel Aviv-Yaffo, Tel-Aviv 6818213, Israel; 3 Department of Psychology, Bar Ilan University, Ramat-Gan 5290002, Israel; ohayonso@biu.ac.il; 4 Bariatric Surgery Unit, General Surgery Division, The Tel-Aviv Sourasky Medical Center, Tel-Aviv 6423906, Israel; subhia@tlvmc.gov.il

**Keywords:** bariatric surgery, body image dissatisfaction, cross-lagged, analysis, gender, psychological distress, suicidal risk

## Abstract

Objectives: This study aimed to develop a gender-specific model to understand the causal relationship between body image dissatisfaction, emotional eating, and suicide risk among bariatric surgery patients. A secondary objective was to evaluate gender differences in the associations between these variables. It was hypothesized that, independent of objective weight loss, body dissatisfaction and emotional eating would lead to increased suicide risk. Methods: A total of 109 participants completed self-report measures of suicidal ideation, body image dissatisfaction, and emotional eating before and after bariatric surgery. Results: Cross-lagged analysis indicated that pre-surgery suicide ideation significantly predicts body dissatisfaction primarily among men, independent of the extent of weight loss. High levels of pre-surgery suicide risk correlated with post-surgery body image dissatisfaction in men. The autoregressive effect of suicide ideation was stronger than that of body dissatisfaction for both genders; however, the latter was stronger among women, indicating that past dissatisfaction levels significantly influenced future dissatisfaction. Conclusions: The complex interplay between gender, body dissatisfaction, emotional eating, and suicide risk warrants further research.

## 1. Introduction

Over the past few decades, obesity has emerged as a global epidemic and a significant public health challenge [1,2,3]. It affects approximately 13% of the world’s population, encompassing a staggering 600 million individuals worldwide [4]. Obesity is closely associated with numerous physical comorbidities, such as type II diabetes, cancer, and cardiovascular diseases [5].

Bariatric surgery has emerged as an evidence-based treatment for obesity, demonstrating its efficacy in reducing body weight and addressing related comorbidities [6]. However, recent studies have suggested that while bariatric surgery may be effective in reducing physical comorbidities, it may not necessarily alleviate aspects of psychological distress, including the risk of suicide [7]. A meta-analysis examining the risk of self-harm and suicide after bariatric surgery revealed an increased risk within the same population (with an odds ratio of 1.9) and in a comparison to matched controls (with an odds ratio of 3.8) [6]. The underlying reasons for this association are not yet fully understood, but likely involve not only physiological processes and behavioral factors but also psychological characteristics. It is, consequently, crucial to gain a comprehensive understanding of the psychosocial mechanisms associated with suicide risk among individuals who have undergone bariatric surgery. Suicidal behavior (among other adverse outcomes) has been linked to prolonged psychological distress [8,9,10]. An increased risk of psychological distress among bariatric surgery patients has been found to be associated with high levels of preoperative body image dissatisfaction, encompassing negative thoughts, beliefs, and feelings regarding one’s body [10]. Additionally, as an expression of prolonged psychological distress, suicidal risk has been found related to emotional eating, namely, the tendency to eat when experiencing negative affects [11,12]. Individuals with obesity may engage in maladaptive emotional eating as a means of soothing themselves and regulating negative and unwanted emotions, thus creating a circle of discontent (CODT) [13,14,15].

Existing findings based on data from North America and Israel indicate a consistent link between suicide risk, body image dissatisfaction, and emotional eating both before and after bariatric surgery, with similar patterns observed across these two cultures [13,16]. The present study therefore looked to examine the directional relationships between the components of CODT (i.e., suicide risk, body image dissatisfaction, and emotional eating). It assessed these variables before and after bariatric surgery, while controlling for the specific effects of the surgery (e.g., weight loss). Given the ambiguity regarding gender differences in the characteristics of patients undergoing bariatric surgery, this study further explored gender differences in the relationship between suicide risk, body image, and emotional eating. It is noteworthy that women are more frequently involved in surgical weight loss interventions than men [17], due primarily to their tendency to be more concerned about body image. They also exhibit higher levels of depressive symptoms, experience greater perceived stress, and engage in unhealthy habits related to their body image [18,19]. However, findings regarding gender differences in emotional eating tendencies among individuals with obesity remain inconclusive [20]. Moreover, there is controversy regarding differences in suicide rates between men and women after bariatric surgery. Two epidemiological studies based on Pennsylvania registers have reported higher suicide rates among men than women (13.7 vs. 5.2 per 10,000) following bariatric surgery [20,21]. Nevertheless, a Swedish nationwide longitudinal study reported that the standardized mortality ratio for suicide after gastric bypass surgery was higher among females than males [22].

Understanding the complex relationships between body dissatisfaction, emotional eating, and suicide risk is crucial, especially considering the significant influence of cultural and societal norms on these dynamics. Several key gaps remain unaddressed. The directionality of these relationships is unclear; for instance, it is not known whether body dissatisfaction drives emotional eating and suicide risk or if heightened suicide risk increases dissatisfaction. Gender-specific differences are also underexplored. For example, societal norms around masculinity may lead men to suppress concerns about body image, while cultural beauty standards often result in women experiencing greater preoccupation with body image. Additionally, the evolution of these relationships over time, particularly after bariatric surgery, remains poorly understood. Clarifying the causal pathways and understanding gender-specific differences can enhance psychological care for bariatric surgery patients, ultimately promoting better mental health outcomes and long-term well-being. For instance, men may benefit from strategies that address suppressed body dissatisfaction and challenge traditional notions of masculinity, while women may require support focused on reducing body image-related stress driven by societal pressures. To address these gaps, the present study aims to explore the causal relationships between suicide risk, body image dissatisfaction, and emotional eating among bariatric surgery patients, using psychological variables measured at two time points: before and after the surgery. The primary objective of this study was to develop a gender-specific causal model, drawing upon the theoretical framework of CODT, to investigate the relationships between body image dissatisfaction, emotional eating, and suicide risk among individuals who have undergone bariatric surgery. The study’s main goal is to identify individuals who may be at an increased risk of suicide. This in turn may encourage the development of customized interventions that can enhance coping strategies and overall well-being across diverse cultural contexts.

Our hypothesis therefore posits that, regardless of objective weight loss, both body image dissatisfaction and emotional eating will contribute independently to an elevated risk of suicide. Given the limited existing research on gender differences in the interactions between the variables within the CODT, we conducted an exploratory analysis to examine potential gender disparities in the relationships between body image dissatisfaction, emotional eating, and suicide risk.

## 2. Methods

### 2.1. Study Design Procedure and Ethics

This longitudinal study, which involved gathering psychosocial data from individuals undergoing bariatric surgery, both before and after the procedure, was conducted in the United States and Israel from 2015 to 2017. Since culture is a dynamic construct that shapes social context, relationships, attitudes, and behaviors, testing the suggested causal model in two distinct cultural settings—an urban Israeli population and a rural Upstate New York population—offers valuable insights. While differences in obesity prevalence and cultural meanings of eating exist, shared Western values and weight-related stigmatization emphasize the importance of this comparison for tailoring psychological interventions and advancing personalized care for bariatric surgery candidates [13].

The research obtained approval from institutional review boards at university-affiliated bariatric centers in the United States [masked for review] (NY, Project #1074, original approval date: 7 January 2015) and Israel [masked for review] (0511-13 TLV, original approval date: 27 January 15).

In the United States, patients qualified for the study upon receiving surgical approval from the bariatric team. The study’s research assistant interviewed potential participants, secured informed consent, and ensured completion of the pre-surgery questionnaire. Post-surgery, a research assistant either administered the follow-up questionnaires during a scheduled clinic visit or mailed them to the patient’s home with a prepaid return envelope post-operation (average time: 14.7 months, SD: 4.4). Weight measurements were extracted from patients’ medical records, selecting the follow-up weight measurement closest to when the questionnaire was completed.

In Israel, participants completed a self-report questionnaire one week before surgery during their preoperative assessment. Following the procedure, a research assistant reached out to patients via phone or mail (average time: 11.6 months, SD: 4.8, range: 3–23) to complete the self-report questionnaires and provide self-reported weights.

Participants’ anonymity was protected through data de-identification, secure storage, and the separation of identifying information from research data. To ensure their psychological well-being, participants were informed about access to counseling services and provided with clear options to withdraw from the study at any time.

As outlined in the introduction, the study’s objective involved combining the two samples from the United States and Israel for analysis purposes.

### 2.2. Participants

Of the 250 participants assessed before the bariatric surgery, 109 (44%) also completed the post-surgery assessment and were included in the study. The sample included 79 (72.5%) women and 30 men (27.5%). Among the participants, 49 individuals (45.4%) had an educational attainment of elementary school or lower, 31 (28.7%) had completed high school, 15 (13.9%) had finished vocational school, and 13 (12.0%) had attained at least one academic degree. In terms of religious affiliation, 65 participants (61.9%) identified as Jewish, 30 (28.6%) as Christian, 2 (1.9%) as Muslim, and 8 (7.6%) as belonging to other religions. Geographically, 73 participants (67%) were from Israel, and 36 (33%) were from the United States. The majority of the participants (77 [79%]) underwent a gastric sleeve operation, 24 (22%) underwent gastric bypass, and 7 (6%) underwent lap band surgery. The type of bariatric surgery was unknown for one participant.

### 2.3. Measures

Socio-demographic data. Participants reported demographic information such as gender, age, height, weight, and education. The body mass index was calculated as weight (kg) divided by the square of height (m) (kg/m^2^). Percent excess weight loss (%EWL) was calculated by assuming normal body weight as a BMI of 25 kg/m^2^, with a change in BMI divided by preoperative BMI–25 = % BMI loss [23].

Body Shape Questionnaire-8C (BSQ-8C) [24]. The BSQ-8C (The original English versions of these questionnaires were translated into Hebrew in previous studies (for details see [13])) is an 8-item questionnaire designed to evaluate participants’ dissatisfaction with the shape of their body. Each item—e.g., Have you felt so bad about your shape that you have cried?—is rated on a 6-point scale ranging from 1 (never) to 6 (always), reflecting the individual’s emotional state during the previous four weeks. The total score is the sum of the 8 items, ranging from 8 to 48, with higher scores indicating higher levels of distress. Internal consistency of the BSQ-8C in the current study was satisfactory (Cronbach’s alpha for Israel = 0.82; United States = 0.77).

Emotional Eating Scale (EES) [25]. The EES is a 25-item scale designed to assess the tendency of individuals to eat in response to negative emotional stimuli—e.g., “sad”, “guilty”, “bored”—with three subscales of anger/frustration, anxiety, and depression. A total score can also be obtained by combining all subscales. Each item is rated on a 5-point scale indicating the desire to eat, ranging from 1 (no) to 5 (overwhelming urge). The 25 items are averaged to obtain a total score that ranges from 1 to 5, with higher scores indicating a greater urge to eat in response to negative mood states. The internal consistency of the EES total in the current study was high (Cronbach’s alpha Israel = 0.95; United States = 0.95).

Suicidal Behaviors Questionnaire-Revised (SBQ-R) [26]. The SBQ-R is a brief 4-item self-report measure of suicidal thoughts and attempts; it includes questions regarding previous suicide attempts, frequency of suicidal ideation, previous suicidal communication, and the subjective likelihood of a future suicide attempt. The scale is obtained as the sum of the items ranging from 3 to 18, with higher scores indicating an increased suicide risk. The internal consistency of the SBQ-R in the current study was satisfactory (Cronbach’s alpha for Israel = 0.76; United States = 0.71).

### 2.4. Statistical Analysis

Statistical analyses were performed using R 4.1.2 [27]. Due to missing values, multiple imputation was conducted using the Amelia II package for R [28] based on five different imputed datasets. Prior to subsequent analyses, we tested whether the missing data met the Missing Completely at Random (MCAR) assumption using the naniar package (1.0.0) [29], as proposed by Little [30]. The results indicated that the missing data met the MCAR assumption (χ^2^ (24) = 16.90, *p* = 0.85), suggesting that there was no significant pattern of missing values. Finally, a path analysis was conducted using semTools (0.5-5) [31]. EWL was included as a covariate in the model, as this is a commonly used measure of the objective success of the surgery. By including this covariate, we test for the unique effect of body image, controlled for the surgery outcome.

Although related, all analyses reported herein are separate and unique from the other investigations of this dataset.

## 3. Results

Table 1 presents the means, standard deviations, and bivariate analysis of the study variables.

**Figure 1:**
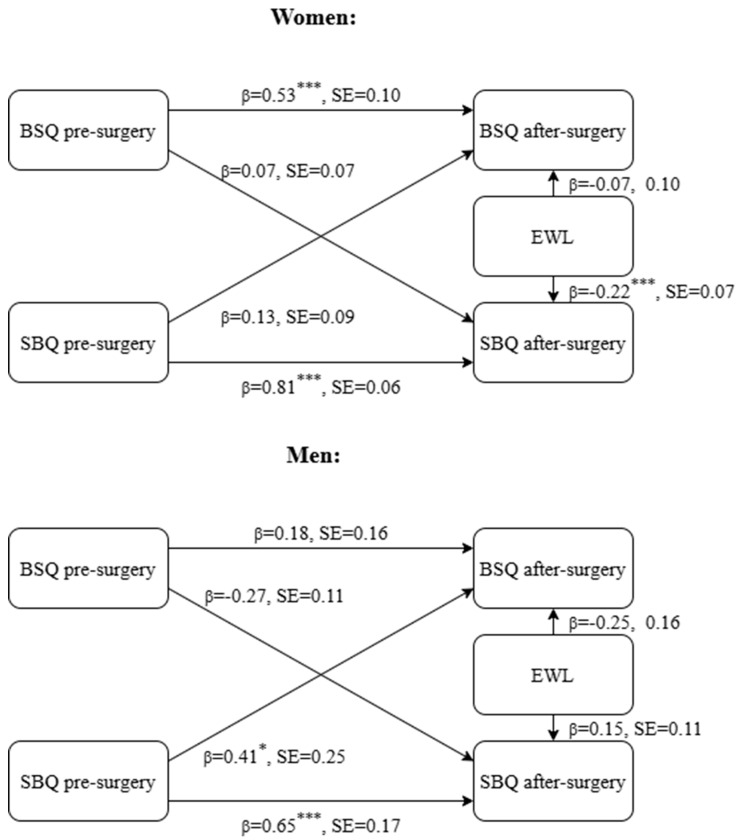
Coefficients and standard error values from cross-lagged, multi-group analysis. Note: *: *p* < 0.05; ***: *p* < 0.001.

To evaluate the research hypotheses, a cross-lagged multi-group analysis was conducted with EES, SBQ, BSQ, and EWL scores. However, as EES scores did not significantly predicate other variables in the model, we repeated the analysis with BSQ, SBQ, and EWL (as covariates) scores. Figure 1 presents the main elements of this model with unstandardized coefficients and standard error values. As Figure 1 suggests, the autoregressive effect of suicide risk was significant for both women and men (women: β = 0.81, SE = 0.06, *p* < 0.001; men: β = 0.65, SE = 0.17, *p* < 0.001). However, the autoregressive effect for body satisfaction was significant only for women (women: β = 0.53, SE = 0.10, *p* < 0.001; men: β = 0.18, SE = 0.16, *p* = 0.31). Furthermore, gender differences were observed in the lagged effects (χ^2^*_diff_* (2) = 3.51, *p* = 0.02). Among men, a significant cross-lagged effect was found (χ^2^*_diff_* (1) = 7.97, *p* = 0.01), so while suicide risk significantly predicted body satisfaction (β = 0.41, SE = 0.25, *p* = 0.02), body satisfaction did not significantly predict suicide risk after controlling for autoregressive effects (β = −0.27, SE = 0.11, *p* = 0.10). Among women, neither the lagged effects of suicide risk on body satisfaction and body satisfaction on suicide risk were significant (β = 0.13, SE = 0.09, *p* = 0.19; β = 0.07, SE = 0.07, *p* = 0.29, respectively) and they were not significantly different from each other (χ^2^*_diff_* (1) = 0.32, *p* = 0.57).

In addition, as Figure 1 suggests, for both women and men, the autoregressive effect of suicide risk (β = 0.81, SE = 0.06, *p* < 0.001; β = 0.65, SE = 0.17, *p* < 0.001, respectively) was greater than the autoregressive effect of body satisfaction (χ^2^*_diff_* (2) = 5.06, *p* = 0.01; β = 0.53, SE = 0.10, *p* < 0.001; β = 0.18, SE = 0.16, *p* = 0.31, respectively).

## 4. Discussion

The main objective of the present study was to develop a gender-specific causal model that examines the relationships between body image dissatisfaction, emotional eating, and suicide risk among bariatric surgery patients. We hypothesized that, irrespective of objective weight loss, both body image dissatisfaction and emotional eating would contribute to an increased suicide risk. However, contrary to our hypothesis, we found that high levels of suicide risk before bariatric surgery were associated with post-surgery body image dissatisfaction among men, regardless of the extent of excess weight loss (EWL).

This finding aligns with the conclusions of one systematic review and meta-analysis [32], which suggested a connection between depression and body image dissatisfaction in men. The authors highlighted the need for longitudinal research to better understand the causal relationships between these variables. Our findings indeed indicate that, at least among men undergoing bariatric surgery, suicidal thoughts may be the underlying cause of body image dissatisfaction. One possible explanation is that men are socialized to avoid addressing their body image concerns [33]. The bariatric surgery procedure may compel men to confront and engage with their bodies, leading highly distressed individuals with suicidal thoughts to manifest their negative preoccupation through body image dissatisfaction [34]. Furthermore, it can be suggested that men experience more body image problems, which are influenced by maladaptive perfectionism mediated by psychological well-being [35].

In contrast, women already tend to be preoccupied with their body image when facing surgery [36] and, therefore, suicidal risk may not necessarily be manifested through body image dissatisfaction.

The autoregressive effect of suicidal risk was found to be stronger than the autoregressive effect of body image dissatisfaction for both genders. It may be argued that underlying affective factors contributing to suicide risk, such as depression, anxiety, and feelings of hopelessness, tend to be more stable over time [37] than factors influencing body image dissatisfaction. Body image dissatisfaction, on the other hand, is more susceptible to fluctuations influenced by various external factors like weight loss, dietary changes, and appearance [38]. It may thus be cautiously suggested that while bariatric surgery can shape and redirect one’s perception of their body [39], it may inadvertently overlook the issue of depression or self-deprecation, operating under the assumption that these factors will automatically improve as a result of the surgery.

Among women, the autoregressive effect for body dissatisfaction was stronger than the same effect among men. This may reflect the profound significance attributed to body image in women due to societal pressures and beauty standards that emphasize thinness and physical appearance [40]. Men, on the other hand, may be less influenced by societal expectations in this regard, resulting in a weaker autoregressive effect for body satisfaction. Recent meta-analyses on gender differences in body dissatisfaction have consistently found that women generally report higher levels of body dissatisfaction than men [41]. This gender difference has been observed across various countries, cultures, and age groups. Thus, women may be more prone to experiencing fluctuations in body satisfaction based on past experiences, leading to a stronger autoregressive effect.

This study highlights the importance of gender-specific interventions in the context of bariatric surgery patients. Distressed men may benefit from targeted interventions that address the interplay between body image concerns and negative self-evaluations, both of which are often exacerbated by rigid gender norms discouraging emotional expression. Sexism reinforces the stigma around male vulnerability, framing body dissatisfaction as incompatible with traditional masculinity, leading many men to suppress insecurities [42]. Open discussions during the post-surgery period, combined with interventions to reduce stigma around vulnerability and address the connection between body dissatisfaction and negative self-evaluations through cognitive-behavioral techniques, could significantly enhance both physical and emotional well-being among men [33]. For women, targeted programs that foster self-compassion and resilience could help to mitigate the impact of societal pressures on body image [38,40]. Equipping clinicians with training to implement these tailored strategies in routine practice would improve the practical application of these findings, effectively addressing body image dissatisfaction and suicide risk in bariatric surgery patients. Additionally, educating healthcare providers about the psychological gender-specific challenges patients may face during their weight loss journey is crucial. By providing comprehensive information on psychological aspects, including body image concerns and suicide risks, both healthcare providers and patients can be empowered to be proactive in providing/seeking support and coping strategies. Finally, the complex interplay between gender, body dissatisfaction, and suicide risk calls for a multidisciplinary approach to the management of bariatric surgery patients. Collaborations between surgeons, psychologists, dietitians, and mental health professionals can ensure holistic care and support throughout the pre- and post-surgery stages.

Despite its contribution, this study has several limitations. First, the relatively small sample size of 109 participants limits the study’s statistical power, particularly for complex analyses; this may reduce the reliability of subgroup comparisons and the findings should therefore be interpreted with caution. Second, the study sample was composed predominantly of women (72.5%), potentially introducing gender bias and limiting the generalizability of the results to men. Third, the study did not specifically examine the effects of different types of bariatric surgery procedures on the studied variables. The majority of participants underwent gastric sleeve operations, while a smaller proportion had gastric bypass or lap band surgery, but the potential influence of the different surgical procedures on the relationships between body image dissatisfaction, emotional eating, and suicide risk was not directly explored. Fourth, the data were collected between 2015 and 2017, and changes in cultural norms, medical practices, or psychosocial factors since then could influence how the findings relate to current populations. Fifth, the high attrition rate, with less than 50% of participants completing the post-surgery psychological assessments, may have introduced selection bias and could limit the generalizability of the findings. Sixth, there were methodological differences in data collection between the U.S. and Israel, including timing, follow-up methods, and weight measurement. These variations may introduce biases. However, combining data from two distinct contexts broadens the study’s scope, offering insights across diverse populations. Finally, the study was conducted in the United States and Israel, which may limit the generalizability of the findings to other cultural contexts. Cultural factors can influence body image, emotional eating patterns, and suicide risks, and therefore, the findings may not apply universally. Future studies should aim to explore the observed gender differences in greater depth by examining the underlying mechanisms driving these disparities. Longitudinal research with larger, more gender-balanced samples could help to clarify causal relationships between body image dissatisfaction, emotional eating, and suicide risk over time. Additionally, investigating how various bariatric procedures differentially impact these psychosocial variables may provide valuable insights for tailoring interventions and optimizing clinical outcomes. Future research should also consider diverse cultural backgrounds to enhance the external validity of the results.

## 5. Conclusions

Overall, this study emphasizes the importance of developing effective interventions to address mental health issues among bariatric surgery patients related to both body image and suicide ideation. The observed autoregressive effects of body satisfaction and suicide ideation highlight the value of longitudinal studies in capturing the dynamic relationships between these variables over time. This study opens up avenues for future research to further explore the complex interplay between body satisfaction, suicide ideation, and other related factors. Investigating these relationships across diverse populations, settings, and cultural contexts could help to refine our understanding and contribute to the development of more targeted and effective interventions. The use of experimental or longitudinal designs might also provide further insight into the causal nature of the relationships between body image dissatisfaction, emotional eating, and suicide risk.

## Figures and Tables

**Table 1:** Descriptive statistics and bivariate analysis of the study variables.

	M (SD)	1	2	3	4	5	6	7
1. SBQ score pre-surgery	3.59 (1.46)							
2. SBQ score post-surgery	3.62 (1.5)	0.80 **						
3. EES score pre-surgery	2.04 (0.79)	0.11	−0.02					
4. EES score after-surgery	1.84 (0.82)	0.04	0.06	0.45 **				
5. BSQ score pre-surgery	25.26 (9.95)	0.19	0.16	0.28 **	0.18			
6. BSQ score after-surgery	19.26 (9.41)	0.27 **	0.34 **	0.09	0.25 *	0.48 **		
7. Age	44.91 (11.7)	−0.18	−0.23 *	−0.08	0.01	−0.12	−0.1	
8. EWL	0.67 (0.31)	0	−0.16	0.20 *	−0.03	0.02	−0.12	−0.21 *

*: *p* < 0.05; **: *p* < 0.01; Abbreviations: SBQ, Suicidal Behaviors Questionnaire; EES, Emotional Eating Scale; BSQ, Body Shape Questionnaire-8C31; EWL, Excess Weight Loss.

## Data Availability

The data generated during and/or analyzed during the current study are not publicly accessible in order to uphold the privacy assurances made to participants. However, they can be obtained from the corresponding author upon reasonable request.

## References

[1] Franco J.V.A., Ruiz P.A., Palermo M., Gagner M. (2011). A Review of Studies Comparing Three Laparoscopic Procedures in Bariatric Surgery: Sleeve Gastrectomy, Roux-En-Y Gastric Bypass and Adjustable Gastric Banding. Obes. Surg..

[2] Barbagallo C., Finucane M., Stevens G., Cowan M., Danaei G., Lin J., Paciorek C., Singh G., Gutierrez H., Lu Y. (2011). National, Regional, and Global Trends in Body-Mass Index since 1980: Systematic Analysis of Health Examination Surveys and Epidemiological Studies with 960 Country-Years and 9? 1 Million Participants. Lancet.

[3] Bray G.A. (2004). Medical Consequences of Obesity. J. Clin. Endocrinol. Metab..

[4] World Health Organization (2021). Obesity and Overweight.

[5] Guh D.P., Zhang W., Bansback N., Amarsi Z., Birmingham C.L., Anis A.H. (2009). The Incidence of Co-Morbidities Related to Obesity and Overweight: A Systematic Review and Meta-Analysis. BMC Public Health.

[6] Sarwer D.B., Dilks R.J., Spitzer J.C., Cash T., Smolak L. (2011). Weight Loss and Changes in Body Image. Body Image: A Handbook of Science, Practice and Prevention.

[7] Yen Y.C., Huang C.K., Tai C.M. (2014). Psychiatric Aspects of Bariatric Surgery. Curr. Opin. Psychiatry.

[8] Gollust S.E., Eisenberg D., Golberstein E. (2008). Prevalence and Correlates of Self-Injury among University Students. J. Am. Coll. Health.

[9] Bayram N., Bilgel N. (2008). The Prevalence and Socio-Demographic Correlations of Depression, Anxiety and Stress among a Group of University Students. Soc. Psychiatry Psychiatr. Epidemiol..

[10] Kisch J., Leino E.V., Silverman M.M. (2005). Aspects of Suicidal Behavior, Depression, and Treatment in College Students: Results from the Spring 2000 National College Health Assessment Survey. Suicide Life-Threat. Behav..

[11] Crow S., Eisenberg M.E., Story M., Neumark-Sztainer D. (2008). Are Body Dissatisfaction, Eating Disturbance, and Body Mass Index Predictors of Suicidal Behavior in Adolescents? A Longitudinal Study. J. Consult. Clin. Psychol..

[12] Miotto P., De Coppi M., Frezza M., Preti A. (2003). Eating Disorders and Suicide Risk Factors in Adolescents: An Italian Community-Based Study. J. Nerv. Ment. Dis..

[13] Geller S., Levy S., Goldzweig G., Hamdan S., Manor A., Dahan S., Rothschild E., Stukalin Y., Abu-Abeid S. (2019). Psychological Distress among Bariatric Surgery Candidates: The Roles of Body Image and Emotional Eating. Clin. Obes..

[14] Appelhans B.M., Whited M.C., Schneider K.L., Oleski J., Pagoto S.L. (2011). Response Style and Vulnerability to Anger-Induced Eating in Obese Adults. Eat. Behav..

[15] Marks D.F. (2015). Homeostatic Theory of Obesity. Health Psychol. Open.

[16] Geller S., Levy S., Hyman O., Jenkins P.L., Abu-Abeid S., Goldzweig G. (2021). Preoperative Body-Related Emotional Distress and Culture as Predictors of Outcomes of Bariatric Surgery. Eat. Weight. Disord..

[17] Mousapour P., Tasdighi E., Khalaj A., Mahdavi M., Valizadeh M., Taheri H., Hosseinpanah F., Barzin M. (2021). Sex Disparity in Laparoscopic Bariatric Surgery Outcomes: A Matched-Pair Cohort Analysis. Sci. Rep..

[18] Lipowska M., Lipowski M., Olszewski H., Dykalska-Bieck D. (2016). Gender Differences in Body-Esteem among Seniors: Beauty and Health Considerations. Arch. Gerontol. Geriatr..

[19] Sabinsky M.S., Toft U., Raben A., Holm L. (2007). Overweight Men’s Motivations and Perceived Barriers towards Weight Loss. Eur. J. Clin. Nutr..

[20] Smith J., Ang X.Q., Giles E.L., Traviss-Turner G. (2023). Emotional Eating Interventions for Adults Living with Overweight or Obesity: A Systematic Review and Meta-Analysis. Int. J. Environ. Res. Public Health.

[21] Konttinen H., Sjöholm K., Jacobson P., Svensson P.-A., Carlsson L.M.S., Peltonen M. (2021). Prediction of Suicide and Nonfatal Self-Harm After Bariatric Surgery: A Risk Score Based on Sociodemographic Factors, Lifestyle Behavior, and Mental Health: A Nonrandomized Controlled Trial. Ann. Surg..

[22] Colarusso L., Serafini M., Lagerros Y.T., Nyren O., La Vecchia C., Rossi M., Ye W., Tavani A., Adami H.-O., Grotta A. (2017). Dietary Antioxidant Capacity and Risk for Stroke in a Prospective Cohort Study of Swedish Men and Women. Nutrition.

[23] Brethauer S.A., Kim J., El Chaar M., Papasavas P., Eisenberg D., Rogers A., Ballem N., Kligman M., Kothari S., ASMBS Clinical Issues Committee (2015). Standardized Outcomes Reporting in Metabolic and Bariatric Surgery. Obes. Surg..

[24] Evans C., Dolan B. (1993). Body Shape Questionnaire: Derivation of Shortened “Alternate Forms”. Int. J. Eat. Disord..

[25] Arnow B., Kenardy J., Agras W.S. (1995). The Emotional Eating Scale: The Development of a Measure to Assess Coping with Negative Affect by Eating. Int. J. Eat. Disord..

[26] Osman A., Bagge C.L., Gutierrez P.M., Konick L.C., Kopper B.A., Barrios F.X. (2001). The Suicidal Behaviors Questionnaire-Revised (SBQ-R): Validation with Clinical and Nonclinical Samples. Assessment.

[27] R Core Team (2021). R: A Language and Environment for Statistical Computing.

[28] Honaker J., King G., Blackwell M. (2011). Amelia II: A Program for Missing Data. J. Stat. Softw..

[29] Tierney N., Cook D. (2023). Expanding Tidy Data Principles to Facilitate Missing Data Exploration, Visualization and Assessment of Imputations. J. Stat. Softw..

[30] Little R. (1988). A Test of Missing Completely at Random for Multivariate Data with Missing Values. J. Am. Stat. Assoc..

[31] Jorgensen T.D., Pornprasertmanit S., Schoemann A.M., Rosseel Y. (2022). SemTools: Useful Tools for Structural Equation Modeling (R Package Version 0.5-6). https://cran.r-project.org/web/packages/semTools/semTools.pdf.

[32] Barnes M., Abhyankar P., Dimova E., Best C. (2020). Associations between Body Dissatisfaction and Self-Reported Anxiety and Depression in Otherwise Healthy Men: A Systematic Review and Meta-Analysis. PLoS ONE.

[33] Sklar E.M. (2017). Body Image, Weight, and Self-Concept in Men. Am. J. Lifestyle Med..

[34] Tantleff-Dunn S., Barnes R.D., Larose J.G. (2011). It’s Not Just a “Woman Thing:” The Current State of Normative Discontent. Eat. Disord..

[35] Hicks R.E., Kenny B., Stevenson S., Vanstone D.M. (2022). Risk Factors in Body Image Dissatisfaction: Gender, Maladaptive Perfectionism, and Psychological Wellbeing. Heliyon.

[36] Mussap A.J. (2007). The Relationship between Feminine Gender Role Stress and Disordered Eating Symptomatology in Women. Stress. Health.

[37] Prenoveau J.M., Craske M.G., Zinbarg R.E., Mineka S., Rose R.D., Griffith J.W. (2011). Are Anxiety and Depression Just as Stable as Personality during Late Adolescence? Results from a Three-Year Longitudinal Latent Variable Study. J. Abnorm. Psychol..

[38] Quittkat H.L., Hartmann A.S., Düsing R., Buhlmann U., Vocks S. (2019). Body Dissatisfaction, Importance of Appearance, and Body Appreciation in Men and Women Over the Lifespan. Front. Psychiatry.

[39] van Hout G.C.M., Fortuin F.A.M., Pelle A.J.M., van Heck G.L. (2008). Psychosocial Functioning, Personality, and Body Image Following Vertical Banded Gastroplasty. Obes. Surg..

[40] Rodgers R.F., Laveway K., Campos P., de Carvalho P.H.B. (2023). Body Image as a Global Mental Health Concern. Glob. Ment. Health.

[41] He J., Sun S., Zickgraf H.F., Lin Z., Fan X. (2020). Meta-Analysis of Gender Differences in Body Appreciation. Body Image.

[42] Mostoller A.M., Mickelson K.D. (2024). Masculinity and Mental Well-Being: The Role of Stigma Attached to Help-Seeking Among Men. Sex Roles.

